# Dual-wavelength UV photofunctionalization of 3D-printed Ti6Al4V porous bone implant enhances osseointegration via adhesion–cytoskeleton–nuclear mechanotransduction

**DOI:** 10.1016/j.mtbio.2025.102581

**Published:** 2025-11-19

**Authors:** Chuan Yin, Yuan Fang, Xiaodong Sun, Zehao Jing, Jingke Fu, Lin Sun, Yan Hou, Eon-Bee Lee, Teng Zhang, Yongtao Wang, Yongqiang Hao

**Affiliations:** a Shanghai Key Laboratory of Orthopaedic Implants, Department of Orthopaedic Surgery, Shanghai Ninth People's Hospital, Shanghai Jiao Tong University School of Medicine, Shanghai, 200011, China; b Shanghai Engineering Research Center of Innovative Orthopaedic Instruments and Personalized Medicine, Clinical and Translational Research Center for 3D Printing Technology, Shanghai, 200011, China; c Department of Plastic and Reconstructive Surgery, Shanghai Ninth People's Hospital, Shanghai Jiao Tong University School of Medicine, Shanghai, 200011, China; d School of Medicine, Shanghai University, Shanghai, 200444, China; e Department of Orthopaedics, Peking University Third Hospital, Beijing, 100191, China; f Department of Aquatic Life Medicine, Pukyong National University, Busan, 48513, South Korea; g Department of Orthopedics, Qilu Hospital of Shandong University, Shandong University, Jinan, 250012, China

**Keywords:** 3D-printed implants, Photofunctionalization, Porous bone implant, Osseointegration, Force-sensing mechanotransduction

## Abstract

Orthopedic implants can be modified with ultraviolet (UV) photofunctionalization to improve osseointegration via force-sensing mechanotransduction in the microenvironment. Meanwhile, few researches have elucidated how UV wavelengths affect the photon-functionalized efficiency to promote synergistic osseointegration. Therefore, three-dimensional (3D)-printed porous Ti6Al4V implants were photofunctionalized with UVC (270 nm; G270), UVA (365 nm; G365), or UV-AC (270 + 365 nm; GU) to investigate the influence of different UV wavelengths on synergistic osseointegration via mechanotransduction. The alkaline phosphatase activity, hydrophilicity, and cytocompatibility of the porous Ti6Al4V implants were considerably higher following UV-AC treatment than following UVA or UVC treatment. Increased bone-implant contact and mineralized (osteoid) bone ratios further showed that UV-AC therapy significantly improved the osteointegration of porous Ti6Al4V implants in an in vivo rabbit condyle defect model. These results suggested that synergistic osteogenic effect of UV-AC irradiation could successfully promote the osseointegration by osteogenic staining and Micro-CT. The surface modification of 3D-printed titanium alloys using multi-wavelength UV functionalization for osseointegration enhancement was supported through adhesion-cytoskeleton-nuclear coupling via force-sensing mechanotransduction in bone tissue microenvironment. This study may be helpful for understanding the photon-functionalized osseointegration of 3D-printed scaffolds via force-sensing mechanotransduction in bone microenvironment.

## Graphical abstract

**Figure:**
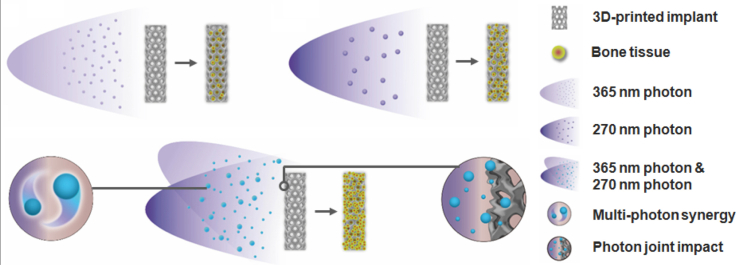

## 1 Introduction

Force-sensitive mechanotransduction plays a crucial role in bone regeneration and repair [[1], [2], [3]]. Biomechanical stimulation can transfer extracellular mechanical stress into the cells and transform these stimuli to biochemical signals into the nuclei to regulate bone regeneration and repair [4]. In particular, mechanical microenvironment can promote the angiogenesis and bone regeneration in large bone defect models [5,6]. In this process, the expression of mechanics-related proteins is very important for osteogenesis. Extracellular mechanics can stimulate the expression of adhesion proteins (integrin, vinculin and talin) and induce network-like cytoskeleton structures (actin and myosin) to improve the mechanical properties of stem cells [7,8]. These mechanics will encourage adhesion-cytoskeleton-nuclear coupling to be associated with YAP/TAZ and nuclear skeleton in the nuclei and promote bone differentiation [9]. Functionalized biomaterials are applied for construction of porous scaffolds to simulate natural bone tissues for their regeneration and repair. The stiffness and topological structure of these scaffolds affect the migration and differentiation of stem cells [[10], [11], [12]]. 3D-printed biological scaffolds have become the potential carriers for bone regeneration and repair to expand their applications in regenerative medicine [13].

Implant failure is a severe complication of orthopedic implant treatment that can result in long-term dependency or disability [14]. With the growing demand for implant therapies, efficient osseointegration has become increasingly crucial in tissue engineering. Ultraviolet (UV) photofunctionalization is a non-coating surface modification technique that can enhance the physicochemical properties and osteogenesis capabilities of Ti6Al4V implants [[15], [16], [17]]. UV irradiation dramatically increases osteogenic cell attachment, retention, and subsequent functional cascades, according to a number of in vitro studies [[18], [19], [20]]. Furthermore, a number of in vivo investigations have demonstrated that bone morphogenesis surrounding titanium implants that have been exposed to UV light is noticeably better than that surrounding untreated control implants [21]. Using a rabbit condyle defect model, we previously showed that UV photofunctionalization can promote faster and more thorough osseointegration of porous 3D-printed Ti6Al4V implants [22].

Although 3D-printed implants have been widely explored in bone repair, the effects of UV photofunctionalization parameters, particularly the UV wavelength, on the osteogenic capability of Ti6Al4V implants have not been thoroughly studied via force-sensitive mechanotransduction. Therefore, determining the optimal parameters and elucidating the underlying mechanisms behind cell nanomechanics of 3D-printed Ti6Al4V implants in osseointegration are essential. In this study, we investigated the effects of UV wavelength (*λ*) on osseointegration by treating 3D-printed porous Ti6Al4V implants with UVC (*λ* = 270 nm), UVA (*λ* = 365 nm), and UV-AC (*λ* = 270 and 365 nm) irradiation. We detected the in vitro effects of UV photofunctionalization at various wavelengths on osteoblast development and cell proliferation. Force-sensitive mechanotransduction was explored by the evaluation of focal adhesion, cytoskeleton distribution and nuclear mechanics. Further, we also constructed a rabbit bone defect model to confirm the osseointegration of 3D-printed implants. Our results revealed the synergistic osteogenic ability of UV-AC on 3D-printed porous Ti6Al4V implants. This may provide some useful information for 3D-printed scaffolds and tissue regeneration via force-sensitive mechanotransduction.

## 2 Materials and methods

### 2.1 Construction of porous Ti6Al4V implants using 3D printing

Porous Ti6Al4V implants with dimensions of 5 mm (diameter) × 6 mm (length), 400 μm pore size, and 73 % porosity were designed and manufactured using electron-beam melting additive manufacturing, according to CAD software (Magics, Materialise, Belgium). This design was selected based on the findings from our prior research, and larger pore sizes were beneficial for the incorporation of bone tissue and vascular structures.

### 2.2 Interfacial photofunctionalization by omnidirectional UV

Prior to UV photofunctionalization, the 3D-printed porous Ti6Al4V implants were autoclave-sterilized and dried for 24 h. Immediately before use, samples underwent 15 min UV photofunctionalization and were handled aseptically thereafter. UV irradiation was delivered in an omnidirectional LED chamber (3 × 3 × 4 cm^3^) assembled with alternating 270 nm and 365 nm emitters to ensure uniform exposure; the porous implants were positioned centrally. Three active UV conditions were used: UVC, 270 nm (G270; irradiance ≥2 mW/cm^2^); UVA, 365 nm (G365; irradiance ≥30 mW/cm^2^); and dual-wavelength UV-AC, 270 + 365 nm (GU). The 15-min exposures correspond to energy doses of ≥1.8 J/cm^2^ (1800 mJ/cm^2^) at 270 nm and ≥27 J/cm^2^ (27,000 mJ/cm^2^) at 365 nm. A non-irradiated control was included (GC, no UV). Group labels (GC, G270, G365, GU) and wavelength names (UVC = 270 nm; UVA = 365 nm) are used consistently in this article.

### 2.3 Assessment of wettability

The static water contact angle was measured using a contact angle goniometer (JC2000C1, POWEREACH, China). A 5 μL deionized water droplet was gently dispensed onto the surface of each sample, and the contact angle was recorded 5 s after dispensing to ensure stabilization. The samples were placed horizontally with the irradiated surface facing upward during measurement. For each group, n = 5 independent samples were tested to calculate the mean ± standard deviation (SD). Statistical differences among groups were analyzed using one-way ANOVA followed by Tukey's post hoc test.

### 2.4 Surface morphogenesis and chemical characterization of porous 3D-printed implants

Before sectioning the specimens with a diamond blade, each sample underwent a UV photofunctionalization treatment. The topography of the cut surfaces was examined with a Hitachi S-4800 field-emission scanning electron microscope (FE-SEM). The acceleration voltage was set at 15 kV. Before the 3D implants were observed by the SEM, the samples were treated with the coating of gold layer to avoid charge accumulation on the 3D-printed implants. To obtain elemental distribution profiles, we utilized energy-dispersive X-ray spectroscopy (EDS; Philips DX-4 system), while chemical bonding states were evaluated through X-ray photoelectron spectroscopy (XPS; Thermo Fisher Scientific 250xi) using monochromatic Al Kα radiation. Crystalline phases were identified via X-ray diffraction (Bruker D8 Focus) utilizing Cu Kα irradiation (λ = 1.5406 Å), with data collected across a 10^°^–80^°^ 2θ range at a scan speed of 0.067°/s.

### 2.5 Biocompatibility evaluation of stem cells

Rat bone marrow mesenchymal stem cells (BMSCs; Lonza, USA) were cultivated on sterilized samples from each group (n = 4) in accordance with a previously described methodology [14]. After 1 h of immersion in the culture medium, the 3D-printed implants were moved to a 24-well plate intended for ultralow attachment. For cellular response evaluation, specimens were immersed in 50 μL growth medium containing 1 × 10^5^ cells/mL and maintained under standard culture conditions (37 °C, 5 % CO_2_) with medium replacement every 72 h. Cellular metabolic activity was quantified at designated intervals (days 3, 7 and 14) using a CCK-8 assay kit (Dojindo Molecular Technologies). Following PBS rinsing, specimens were incubated with 10 % CCK-8 reagent for 180 min at physiological temperature before measuring optical density at 450 nm. Cytoskeletal organization and cell-surface interactions were visualized through F-actin staining coupled with SEM imaging. Viability assessment was conducted via dual fluorescent staining (calcein-AM/PI) and analyzed by the laser confocal microscopy.

### 2.6 Alkaline phosphatase activity measurement

Osteogenic potential was determined through alkaline phosphatase (ALP) activity measurement during differentiation induction using osteogenic supplements (0.5 % ascorbate, 1 % β-glycerophosphate, 0.5 % dexamethasone). ALP enzymatic activity was quantified at days 7 and 14 post-seeding using a commercial assay system (Beyotime Biotechnology) following manufacturer protocols. The samples were washed with PBS 3 times. The fixed cells were soaked in 0.1 wt% naphthol AS-MX phosphate (Sigma) and 0.1 wt% Fast Blue RR salt (Sigma) in 56 mM 2-amino-2-methyl-1,3-propanediol (pH 9.9, Sigma) for 10 min at room temperature. After the samples were washed with PBS 3 times, the 3D-printed implants were observed by an optical microscope to obtain the ALP staining images.

### 2.7 Mechanical testing of 3D-printed implants

To assess the impact of UV photofunctionalization on the mechanical properties of Ti6Al4V implants, we employed a universal testing machine (Landmark, MTS Systems Corp., USA). This analysis focused specifically on the compressive strength before and after UV exposure. Measurements for yield strength, elastic modulus, and compressive strength were conducted on samples (diameter = 10 mm, height = 5 mm; n = 5) at a deformation rate of 1.8 mm/s (108 mm/min).

### 2.8 In vivo osseointegration assessment of 3D-printed implants

To evaluate the osseointegration capacity of porous Ti6Al4V implants in vivo, cylindrical scaffolds were surgically inserted into the bilateral femoral condyles of healthy adult male New Zealand rabbits under approval of the Peking University Health Science Center Animal Ethics Committee (protocol LA2014214) and in accordance with the Animals (Scientific Procedures) Act 1986. Anesthesia was induced with ketamine (50 mg/kg, intramuscular), with supplemental isoflurane (1–2 % inhalational) provided as needed. Peri-operative analgesia was administered according to institutional veterinary guidance to minimize pain and distress, and animals were monitored at least twice daily (appetite, activity, wound status, mobility). Postoperative penicillin was administered as prophylaxis against infection. Humane endpoints were predefined (≥15 % body-weight loss, persistent anorexia >24 h, severe lameness, or wound dehiscence/infection unresponsive to treatment). Exclusion criteria were prospectively defined (implant malposition, intra-operative fracture, postoperative infection). Any animals meeting these criteria would have been excluded and replaced; adverse events were prospectively recorded. In vivo sequential fluorescent labeling (calcein green and tetracycline at predefined intervals) was performed to track bone formation, followed by euthanasia and sample harvesting for histology, fluorescence microscopy, micro-CT, and mechanical testing.

### 2.9 Micro-computed tomography analysis

Using high-resolution micro-computed tomography (micro-CT; Inveon, Siemens Medical Solutions, USA), bone integration and growth were quantitatively evaluated. Inveon Acquisition Workplace was used for the reconstruction of the micro-CT images. Hounsfield units (HU) were divided to separate metal implants and soft tissue from newly formed bone. The 1000–2250 HU range was considered the bone phase. Within the workstation, two critical regions of interests (ROIs) were identified to characterize the development of new bone on the 3D-printed implants. These areas included the intraporous region within the implants and the peri-implant region, extending 500 μm around the porous implants.

### 2.10 Histological and fluorescence analysis

Following euthanasia, samples were prepared for histological examination by fixation, dehydration, and embedding in methyl methacrylate. Fluorescence microscopy was conducted on ground sections to identify bone formation markers at various stages post-implantation. To differentiate implant materials from cartilage, osteoid, and mineralized bone, sections were stained following the Goldner trichrome procedure. Quantitative analysis was carried out using Image-Pro Plus. Bone-implant contact ratio (BICR) and bone ingrowth (BI) were measured in two central longitudinal sections of each block. BI (%) = area fraction of newly formed bone within the intraporous ROI normalized to total pore area; BICR (%) = fraction of implant surface perimeter in direct contact with bone (no intervening gap) within the ROI. We analyzed two central longitudinal sections per specimen; twelve sections per group were measured and averaged per animal before group statistics. In addition, the MSCs were also stained with phalloidin on the surfaces of UV-treated 3D-printed implants to observe their cytoskeleton distribution. The nuclei were stained with DAPI. The fluorescent images were captured by a fluorescent microscope.

### 2.11 Push-out tests

The mechanical bonding between the implants and bone was measured by using push-out tests on a universal testing machine (Landmark, MTS Systems Corp., USA). The force needed to remove the implants was assessed using the samples that were processed to reveal the contact between the implants and bone. This gave a clear indication of the strength for osseointegration.

### 2.12 Western blot

Western blot (WB) was used to evaluate the expression levels of adhesion, cytoskeleton and mechanics-related proteins on the 3D printing implants. For the cells and bone tissues on the 3D-printed implants, the proteins were extracted according to previous protocols. The primary antibodies against Integrin (Integrin-β1/CD29 Rabbit mAb, A2217, ABclonal, China), vinculin (Vinculin Rabbit mAb, A2752, ABclonal, China), YAP (YAP1 Rabbit mAb, A19134, ABclonal, China) and LaminA/C (LaminA/C Antibody, 10298-1-AP, Proteintech, China) were used to incubate the proteins for WB analysis. ImageJ software was used to measure the WB results and define the normalization of proteins of interest to β-actin.

### 2.13 Proteomic analysis

The proteins were extracted from four groups for proteomic analysis. In detail, the samples were washed with PBS and further lysed with Tris-HCl (20 mM) for sonication. After centrifugation, the supernatant was collected to perform protein concentration by a BCA protein kit. LC-MS/MS test was measured by an EASY-nanoLC 1200 system with the Explorise 480 mass spectrometer (Thermo Fisher). Formic acid (FA) and 80:20:0.1 ACN-H_2_O-FA were considered as mobile phases (phase A and phase B). The flow rate was 150 nL/min and the injection volume was 2 μL. The system was set: 6 % B for 0 min, 35 % B for 0–76 min, 35–68 % B for 76–100 min, 68–98 % B for 109-105 min and 98 % B for 105–120 min. Then, the obtained data was detected by Spectronaut software to calculate protein quantification for proteomic analysis.

### 2.14 Statistical analysis

Statistical analysis involved appropriate parametric (Student's t-test) or non-parametric (Mann-Whitney U) testing based on normal distribution assessed by Shapiro-Wilk criteria. All computations were performed using SPSS Statistics 17.0 with *p* < 0.05 significance threshold. Data representation follows the convention of mean ± standard deviation (SD).

## 3 Results

### 3.1 Conceptualization and characterization of 3D-printed implants by UV photofunctionalization

**Fig. 1:**
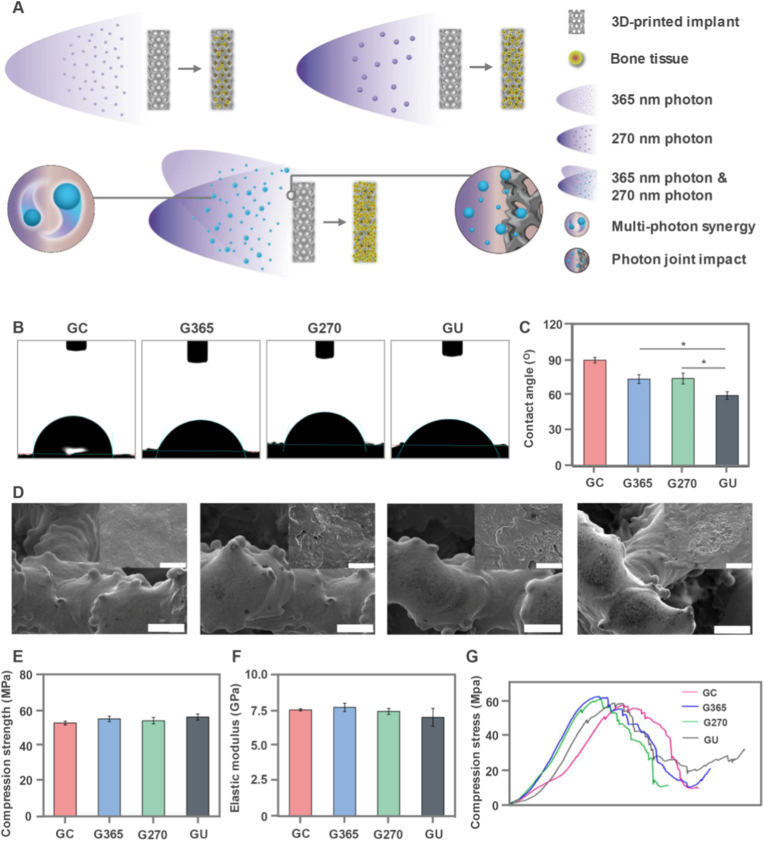
Conceptualization and characterization of 3D-printed Ti6Al4V implants by UV photofunctionalization. (A) Illustration of UV photon functionalization on 3D-printed Ti6Al4V implants by 270 and 365 nm photons to reveal photon joint impact between the interface of 3D-printed implants and bone tissues. (B) Representative water droplet images of contact angle measurement. (C) Water contact angles of four implants. (D) Representative SEM images of four implants. Scale bar: 100 μm. The inserts were the enlarged images of fiber skeleton on the 3D-printed implants. Scale bar: 5 μm. (E) Elastic modulus of 3D-printed implants. (F) Compression strength of 3D-printed implants. (G) Compression stress-strain curves of 3D-printed implants. The data present mean ± SD, n = 5, ∗*p* < 0.05.

The 3D-printed Ti6Al4V implants were conceptualized by the UV photofunctionalization treatment (270 and 365 nm photons) to create the biological analogues of bone tissues via both multi-wavelength synergy and photon joint impact at the interface between the 3D-printed implants and bones (Fig. 1A). The 3D-printed implants were successfully constructed to induce the bone regeneration and repair based on their excellent biocompatibility and biosafety. Especially, the wettability played a crucial role in regulating cell adhesion and spreading on the 3D-printed implants. The water contact angles were measured and the water droplets presented the typical hemispheres, indicating the good wettability on the implants (Fig. 1B). Further, The water contact angles were also calculated to be 87.6° ± 2.8°, 74.1° ± 3.7°, 74.6° ± 4.6° and 60.2° ± 3.2° for GC, G365, G270 and GU, respectively (Fig. 1C). The results showed that GU exhibited the lowest contact angle and had more hydrophilic essence, which was beneficial for cell adhesion and spreading on the implants. Generally, the wettability was closely correlated with the surface microstructure of 3D-printed implants. The microtopography of these implants was observed by SEM to examine their surface morphology (Fig. 1D). Interestingly, all implants displayed network-like scaffolds with fiber skeleton by SEM observation. The enlarged SEM images of fiber skeleton presented the hierarchical roughness structures on GU implants, compared to smooth surface appearance on GC implants. The hierarchical surface morphogenesis of 3D-printed implants determined their wettability by photon joint impact to manipulate cell adhesion and spreading ability on these 3D-printed implants.

The mechanical characteristics of the 3D-printed implants also played the decisive role in bone regeneration and repair, especially for the osseointegration on the interfacial surfaces of the implants. The elastic modulus was monitored to be 6.8–7.6 GPa for all 3D-printed implants (Fig. 1E), showing strong mechanical ability. The compression strength was also measured to exhibit the similar results, ranging from 50 to 60 MPa (Fig. 1F). In addition, compression stress-strain curves were obtained to assume the consistent parameters among four implants (Fig. 1G). These mechanical characteristics indicated that UV photofunctionalization treatment didn't affect elastic modulus and compression strength of 3D-printed implants, which contributed to bone regeneration and repair via photon joint impact. Therefore, the 3D-printed Ti6Al4V implants were well constructed by UV photofunctionalization to manifest excellent wettability, hierarchical surface morphology and robust mechanics.

### 3.2 Evaluation of physicochemical composition by EDS, XRD and XPS analysis

**Fig. 2:**
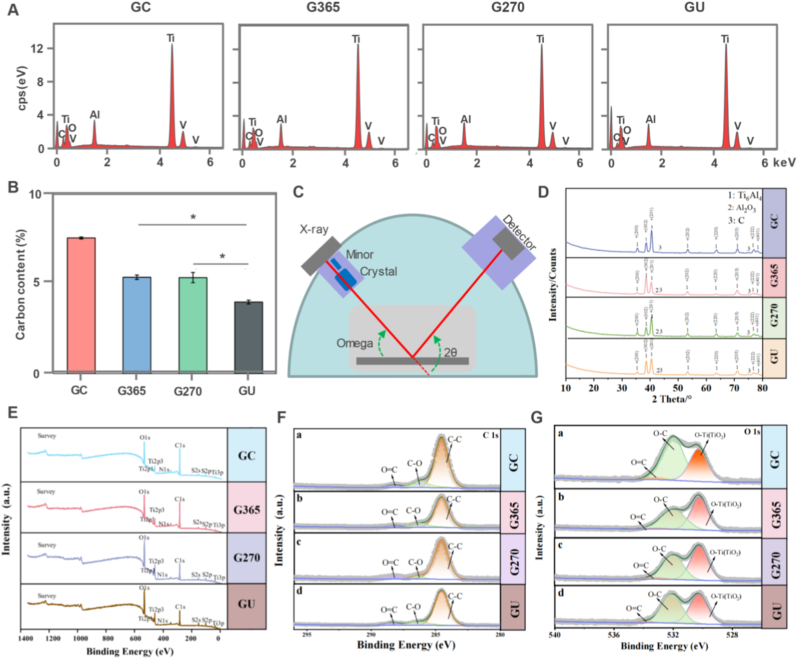
Physicochemical evaluation and elemental composition analysis. (A) EDS characterization spectra of 3D-printed Ti6Al4V implants. (B) Carbon (elemental C) Contents on the inner surfaces of the implants. (C) Illustration of XRD to analyze crystal structure. (D) XRD patterns of the GC, G365, G270, and GU implants. (E) Full XPS spectra. (F) C 1s spectra. (G) O 1s spectra of 3D-printed Ti6Al4V implants. The data present mean ± SD, n = 5, ∗*p* < 0.05.

The physicochemical composition was evaluated to reveal their chemical properties by the UV photofunctionalization on the 3D-printed implants. The EDS spectra showed that all the 3D-printed implants included Ti, Al, V, C and O elements (Fig. 2A). Moreover, carbon content of four implants was detected to present the decreased tendency with the treatments of 365 nm photon, 270 nm photon and both photons (Fig. 2B). The inner surface carbon contents of the G270 and G365 groups do not differ significantly. In addition, XRD could accurately probe the crystal structure of 3D-printed Ti6Al4V implants in biomaterials to explore their role in bone tissue repair (Fig. 2C). The phase composition of the 3D-printed implants both before and after UV photofunctionalization was clarified using XRD (Fig. 2D). The XRD patterns showed several Ti6Al4V diffraction peaks as well as tiny carbon peaks because of the carbon in the titanium matrix. Ti6Al4V was the main phase of 3D-printed implants with characteristic 2*θ* diffraction peaks at 35.921°, 39.010°, 41.166°, 53.921°, 64.677°, 72.030°, 77.923°, and 79.549°, corresponding to the (200), (002), (201), (202), (220), (203), (222), and (401) planes of Ti6Al4V, respectively. The XRD results indicated that the treatment of UV photons didn't change the crystal structure of 3D-printed Ti6Al4V implants.

After UV treatment, the chemical makeup and states of the various components were analyzed via XPS on 3D-printed Ti6Al4V implants. The survey spectra indicated that Ti, C, and O are the main components (Fig. 2E). The C 1s peaks in all samples were deconvolved into three peaks, which represented C–O, O=C, and C–C, respectively, with binding energies of 286.41, 288.41, and 284.82 eV (Fig. 2F). In a similar vein, the O 1s peaks were resolved into three distinct peaks with binding energies at 529.99, 531.89, and 532.99 eV, which corresponded to O–Ti (TiO_2_), O–C, and O=C, respectively (Fig. 2G). These findings indicated that the surface of the 3D-printed porous titanium alloy consisted of both carbon oxide and titanium dioxide, regardless of the UV treatment. Collectively, 3D-printed Ti6Al4V implants were modified by UV photofunctionalization to regulate their carbon content, crystal structure and chemical components via UV photon joint impact.

### 3.3 Cell proliferation and differentiation of biocompatible 3D-printed implants

**Fig. 3:**
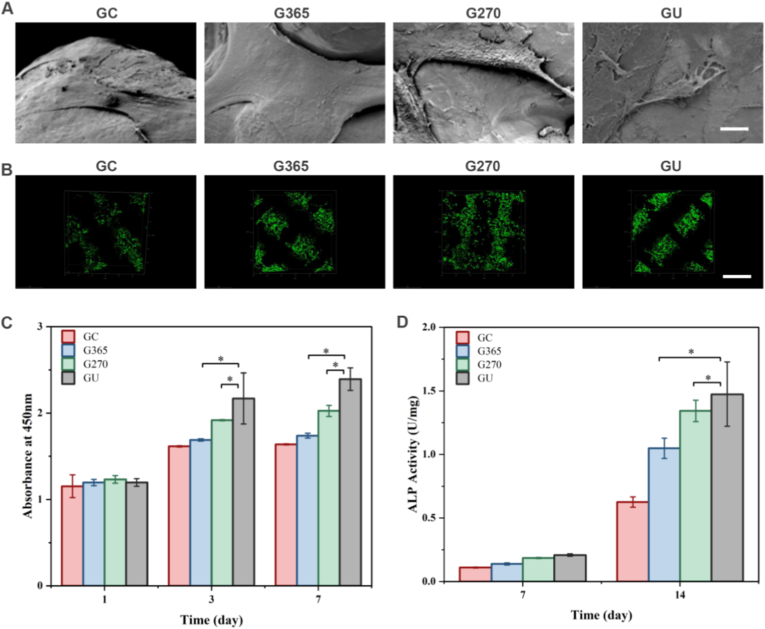
Cell proliferation and differentiation of biocompatible 3D-printed implants. (A) Representative SEM images of rat BMSCs on the 3D-printed implants. Scale bar: 50 μm. (B) Live/dead staining of GC, G365, G270, and GU implants at 3 d. Live and dead cells were stained green and red, respectively. Scale bar: 500 μm. (C) Rat BMSC proliferation on GC, G365, G270, and GU implants at 1, 3, and 7 d. (D) ALP activity of rat BMSCs on GC, G365, G270, and GU implants at 7 and 14 d. The data present mean ± SD, n = 5, ∗*p* < 0.05. (For interpretation of the references to colour in this figure legend, the reader is referred to the Web version of this article.)

3D-printed Ti6Al4V implants could present excellent biocompatibility and biosafety due to good wettability and hierarchical microstructures. Cellular adhesion characteristics and spreading organization at day 7 were documented through combined SEM and fluorescence microscopy evaluation. The SEM images showed that the well-stretched rat BMSCs on the GU, G270, and G365 implants exhibited filiform shapes and excellent adhesion ability (Fig. 3A). Compared to those on the GC implants, the rat BMSCs on the GU, G270, and G365 implants were more evenly distributed with more attached collagen fibers, especially for GU implants. In addition, live/dead staining was used to measure cell viability at 3 days (Fig. 3B). The absence of dead cells (red staining) indicated that rat BMSCs survived on all implants. The cell survival ability was calculated to be more than 98 % among GC, G365, G270 and GU implants (Fig. S1). The cell densities on the GU, G270, and G365 implants were higher than those on the GC implants. Furthermore, in vitro proliferation assessment revealed time-dependent metabolic activity variations among four experimental implants. The GU cohort demonstrated superior proliferative capacity compared to G270 and G365 groups throughout the 7-day observation period, with G270 showing intermediate activity level (Fig. 3C). The differentiation ability of bone tissues was also detected by ALP staining on the 3D-printed implants. Parallel ALP activity analysis identified 14.3 % and 22.7 % reductions in G270 and G365 groups respectively versus GU controls at day 14 (p < 0.05), maintaining consistent intergroup differences (Fig. 3D). Consequently, the proliferation and differentiation of rat BMSCs were managed by UV photon modification on the biocompatible 3D-printed implants.

### 3.4 Cell nanomechanics of 3D-printed implants via adhesion-cytoskeleton-nuclear coupling

**Fig. 4:**
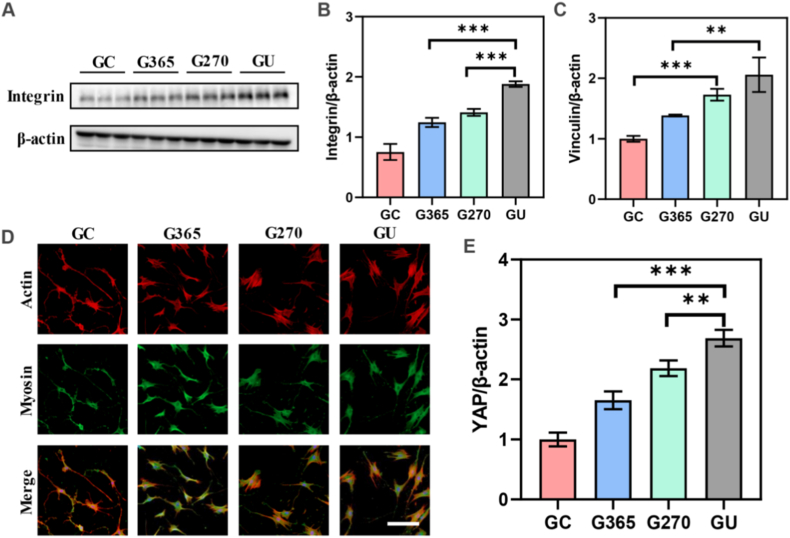
Cell nanomechanics of 3D-printed implants via adhesion-cytoskeleton-nuclear coupling. (A) WB analysis of integrin protein of rat BMSCs on the 3D-printed implants. (B) Expression level of integrin proteins on GC, G365, G270, and GU implants. (C) Expression level of vinculin proteins on GC, G365, G270, and GU implants. (D) Representative images of cytoskeleton structures. Red: Actin; Green: Myosin; Blue: Nuclei. Scale bar: 200 μm. (E) Expression level of YAP proteins on GC, G365, G270, and GU implants. The data present mean ± SD, n = 3, ∗∗*p* < 0.01, ∗∗∗*p* < 0.001. (For interpretation of the references to colour in this figure legend, the reader is referred to the Web version of this article.)

Cell nanomechanics can play a crucial role in regulating force-sensing mechanotransduction in stem cells [23]. The cells perceive the extracellular stimuli from microenvironment to deliver these mechanical signals into cells by adhesion proteins and cationic force-related channels on the cell membrane [24]. Integrin was the key cell adhesion protein to guide cell adhesion behavior on 3D-printed implants. WB analysis was used to detect the expression level of integrin proteins (Fig. 4A). The WB results showed that the expression level of integrin was increased with the photon treatment and GU implants presented the highest integrin expression level (Fig. 4B). Furthermore, the formation of focal adhesion also participated in cell adhesion and spreading ability among four 3D-printed implants. Vinculin, a key protein of focal adhesion, was investigated by WB analysis to reveal its interfacial heterogeneity on different 3D-printed implants (Fig. S2A). The WB data indicated that the expression level of vinculin exhibited an increasing tendency with the photon treatment, which was consistent with integrin expression (Fig. 4C). Thus, Integrin was the key transmembrane protein between extracellular matrix (ECM) and cytoskeleton to regulate nanomechanical behavior for mechanotransduction. They could transmit these external forces into actin cytoskeleton through typical aptamers (talin and vinculin) to activate force-related signal molecules of focal adhesion kinase (FAK), thus affecting cell proliferation and differentiation.

Cell nanomechanical properties were managed via adhesion protein expression of integrin and vinculin to monitor the recombination and distribution of cytoskeletons (microtubules, microfilaments and intermediate fibers) and motor proteins (myosin). Herein, myosin motor protein was stained to reveal its assembly on different photon treatments (Fig. 4D). The actin filaments were also investigated by immunofluorescent staining. The staining photos showed that the cell would form more pseudopod and spread their cytoskeleton on both photon treatments (GU). These cytoskeleton distributions and mechanical signals could be delivered into nuclei to regulate nuclear mechanotransduction. These mechanical stimuli were consistent with downstream MAPK and YAP/TAZ pathways to directly regulate the phosphorylation level of FAK and realize the linear force-nuclear coupling, thus accurately affecting cell migration and differentiation. The YAP was evaluated by WB analysis to investigate the expression level on different photon treatments (Fig. S2B). The WB data exhibited that the expression level of YAP proteins was increased with the UV photon treatments from GC, G365 and G270 to GU (Fig. 4E). These mechanics also impacted the nuclear skeleton-related proteins. To unlock the molecular mechanism of nuclear skeleton-related protein expression, LaminA/C was detected by WB analysis (Fig. S2C). The WB data indicated that LaminA/C presented the gradually increasing expression level with the UV photon treatments (Fig. S2D). Therefore, cell nanomechanics of 3D-printed implants could be mediated by the regulation of adhesion proteins, cytoskeleton and nuclear mechanotransduction via adhesion-cytoskeleton-nuclear coupling.

### 3.5 In vivo histological evaluation of bone formation by 3D-printed implants

**Fig. 5:**
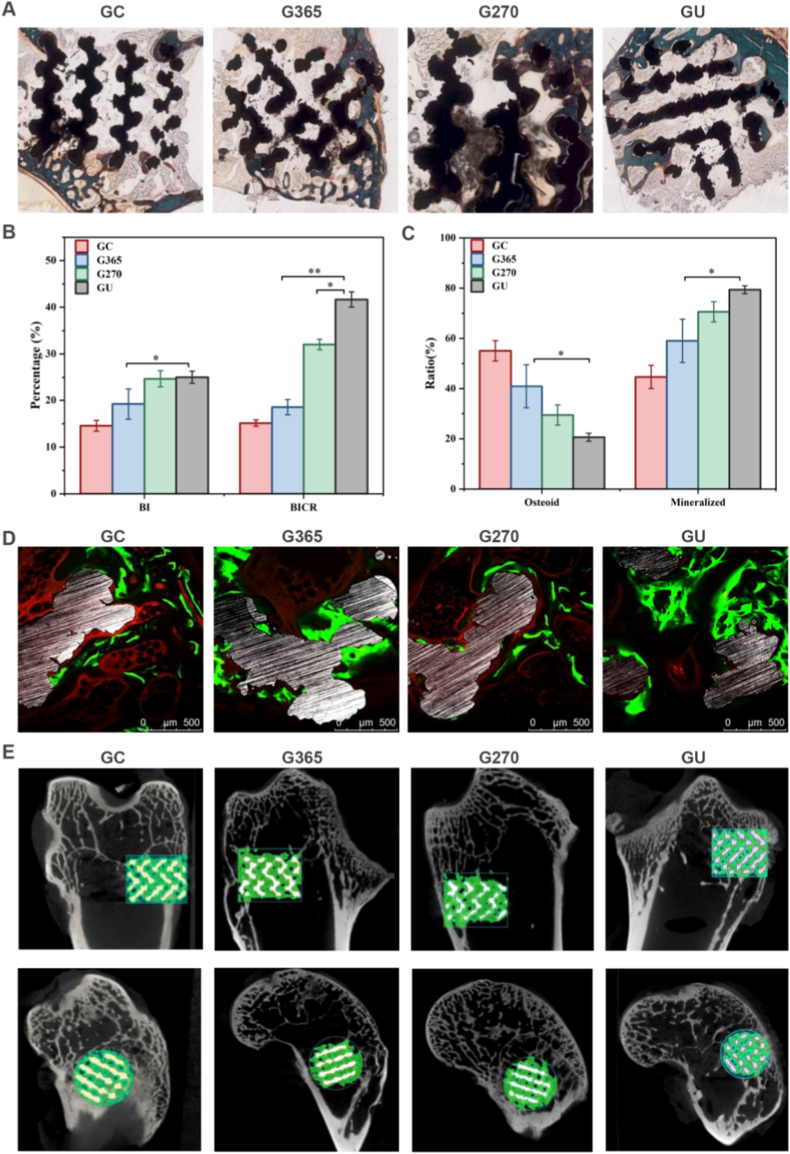
In vivo histological evaluation of bone formation by 3D-printed implants. (A) Representative Goldner trichrome staining images of GC, G365, G270, and GU groups. (B) Percentage of BI (bone ingrowth) and BICR (bone-implant contact ratio) values in the GC, G365, G270, and GU implants. (C) Mineralized bone and osteoid fractions of GC, G365, G270, and GU groups. (D) Representative fluorescent micrographs of osseointegration within the pores of the GC, G365, G270, and GU implants (green and yellow regions denote bone formed at 3 and 7 weeks, respectively, via fluorescence labeling at these timepoints using calcein green and tetracycline, respectively). (E) Micro-CT images of GC, G365, G270, and GU porous implants 8 weeks post-implantation (Top panel: side views; Bottom panel: top views). Peri-implant and intraporous bone was labeled green. The data present mean ± SD, n = 10, ∗*p* < 0.05, ∗∗*p* < 0.01. (For interpretation of the references to colour in this figure legend, the reader is referred to the Web version of this article.)

3D-printed implants were applied to evaluate their in vivo bone formation ability by different UV photon treatments. The degree of osseointegration in each of the four groups was shown in representative histology images (Fig. 5A). Quantitative measurements of BI and BICR (Fig. 5B) and the mineralized bone and osteoid fractions (Fig. 5C) in the ROIs confirmed these histomorphological results. The BI scores for both the GU and G270 groups were significantly elevated compared to the GC group (p < 0.05). Likewise, the BICR values were notably higher in the GU and G270 groups than in the G365 group (p < 0.05), and strikingly greater than those of the G365 group (p < 0.01). As depicted in Fig. 5D, the ratio of mineralized bone was significantly lower in the GC group compared to the GU group (p < 0.01). Furthermore, the GU group showed the signs of early bone growth, often observed as early as three weeks, according to fluorescence labeling.

Further, the bone development was measured using micro-CT analysis to assess how UV photofunctionalization affected the porosity of implants for in vivo osseointegration. The white and green regions of micro-CT images (Fig. 5E) represented the Ti alloy and bone, respectively. The GU, G270, and G365 groups exhibited greater bone formation than the GC group. The micro-CT images displayed the findings of the quantitative examination of the intraporous bone fraction. Compared to the G365 group (66.16 ± 1.52 %), the intraporous bone fraction was significantly higher in the GU (75.53 ± 1.4 %) and G270 groups (74.44 ± 1.9 %) (Fig. S3). Collectively, the compatible 3D-printed implants promoted the osseointegration formation through the regulation of histological and micro-CT analysis.

### 3.6 Biological fixation strength of osseointegration on 3D-printed implants

**Fig. 6:**
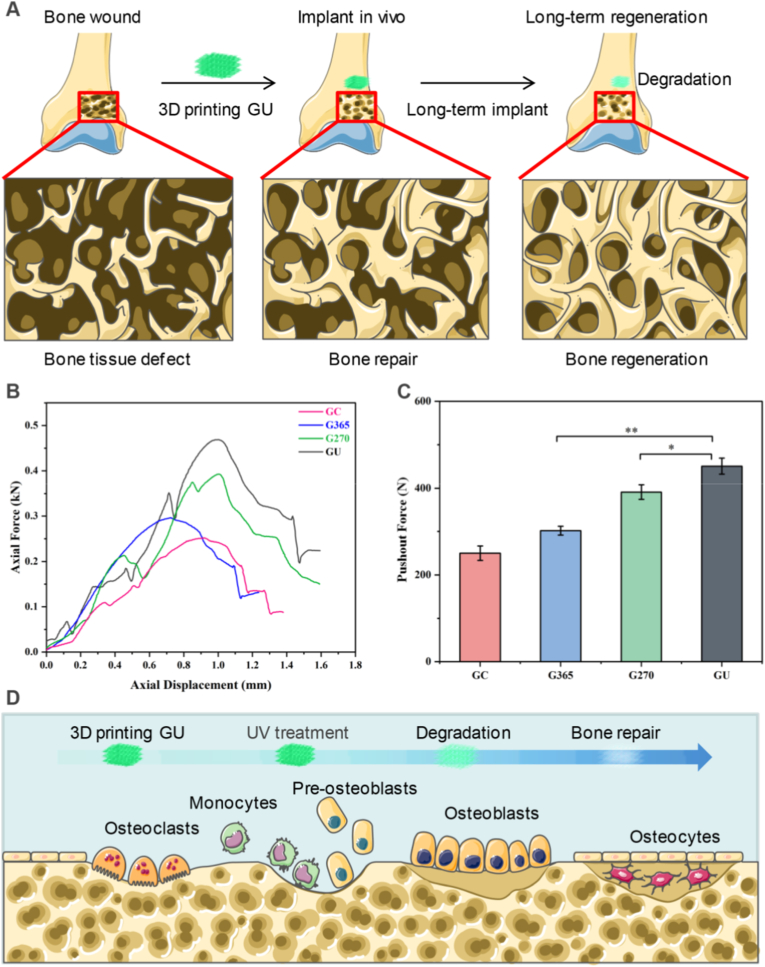
Evaluation of osseointegration fixation strength on 3D-printed implants. (A) Illustration of 3D-printed implants to promote bone tissue repair by long-term bone regeneration. (B) Displacement curves of porous implants 8 weeks post-implantation. (C) Push-out force obtained from the curves of axial force and displacement. (D) Schematics of push-out test of 3D-printed implants from osteoclasts, monocytes, pre-osteoblasts to osteoblasts in osteocytes. The data present mean ± SD, n = 5, ∗*p* < 0.05, ∗∗*p* < 0.01.

3D-printed implants could interact with peripheral bone tissues to stimulate the formation of osseointegration at the interfacial surface of implants and bones (Fig. 6A). When bone wound was induced by bone tissue defect, the 3D-printed implants were embedded into damaged bones in vivo to provoke immune response, which stimulated immune cells to eliminate tissue fragments and release growth factors (BMP, TGF-β and VEGF) for bone tissue repair. Then, rat BMSCs migrated from bone marrow and blood vessels onto the surface of 3D implants and differentiated into osteoblasts. These osteoblasts could secrete bone matrix on the surface of 3D-printed implants to form woven bones, which gradually matured and continued to deposit new bone formation for long-term bone regeneration, thus promoting the mechanical adaptability of bone-implant interface.

Representative displacement curves for the push-out experiments were measured by force-distance analysis (Fig. 6B). The UV-photofunctionalized 3D-printed implants exhibited steeper curves than the GC implant, indicating that a stronger force was required to generate the same displacement. The push-out forces for each group were also compared in different photon treatments (Fig. 6C). The GC group's estimated push-out force was roughly 304.7 ± 29.75 N, while that of the GU group was up to 450.5 ± 18.5 N (*p* < 0.05). The mechanical adaptability between bone and 3D-printed implants was mediated by UV photon treatments. 3D-printed implants could promote osteoclast formation by inducing monocytes and pre-osteoblasts maturation to achieve bone repair in osteocytes (Fig. 6D). Therefore, the 3D-printed implants could improve appropriate osseointegration by the mechanical adaptability between 3D-printed implants and bone tissues via force-related mechanotransduction.

### 3.7 Underlying mechanism of osseointegration via force-sensing mechanotransduction on 3D-printed implants

**Fig. 7:**
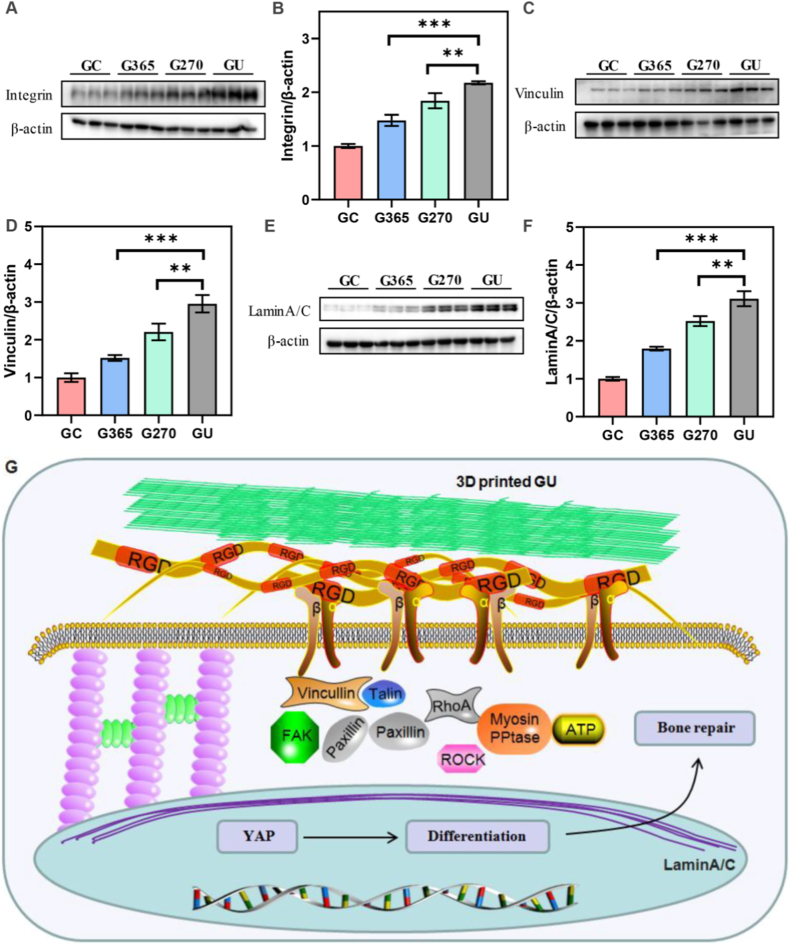
Evaluation of force-sensing mechanotransduction on osseointegration on 3D-printed implants. (A) WB analysis of integrin protein of bone tissues on the 3D-printed implants. (B) Expression level of integrin proteins on GC, G365, G270, and GU implants. (C) WB analysis of vinculin protein of bone tissues on the 3D-printed implants. (D) Expression level of vinculin proteins on GC, G365, G270, and GU implants. (E) WB analysis of LaminA/C protein of bone tissues on the 3D-printed implants. (F) Expression level of LaminA/C proteins on GC, G365, G270, and GU implants. (G) Illustration of 3D-printed implants to transform extracellular stimuli into cells by adhesion-cytoskeleton-nuclear coupling and promote bone repair via YAP and LaminA/C force-sensing mechanotransduction. The data present mean ± SD, n = 3, ∗∗*p* < 0.01, ∗∗∗*p* < 0.001.

To explore the underlying mechanism of osseointegration behind bone tissue defect for mechanical adaptability, force-sensing mechanotransduction was investigated by the adhesion-cytoskeleton-nuclear coupling to reveal their interplay on long-term bone tissue regeneration on 3D-printed implants. Cell adhesion behaviors played a crucial role in sensing extracellular ECM mechanics and transmitted these biophysical stimuli into cells to affect cell force. Integrin was detected by WB analysis to disclose the expression level of adhesion proteins (Fig. 7A). The WB results showed that the expression level was gradually enhanced with the UV photon treatments (GC, G365, G270 and GU) (Fig. 7B). Another adhesion-related protein, vinculin, was also investigated to clarify its expression level by WB analysis (Fig. 7C). The WB data exhibited that the expression level of vinculin presented the increasing tendency with the UV photon treatments (Fig. 7D). Further, these adhesion behaviors could change cytoskeleton distribution and induce these adhesion signals into nuclei to oversee nuclear force-sensing mechanotransduction. YAP was evaluated by WB analysis to probe its force-sensing mechanotransduction on 3D-printed implants (Fig. S4A) and the expression level was elevated when the 3D-printed implants were treated with UV photons, especially for GU group showing the highest level (Fig. S4B). Nuclear skeleton of LaminA/C was also changed by these UV photon treatments on 3D-printed implants and further explored by WB analysis (Fig. 7E). The WB data displayed the lifting results on GC, G365, G270 and GU implants (Fig. 7F). Consequently, the osseointegration behind bone tissue defect on 3D-printed implants was encouraged through adhesion protein formation, YAP signaling activation and nuclear skeleton assembly via nuclear force-sensing mechanotransduction.

### 3.8 Proteomic analysis of force-sensing mechanotransduction on 3D-printed implants

**Fig. 8:**
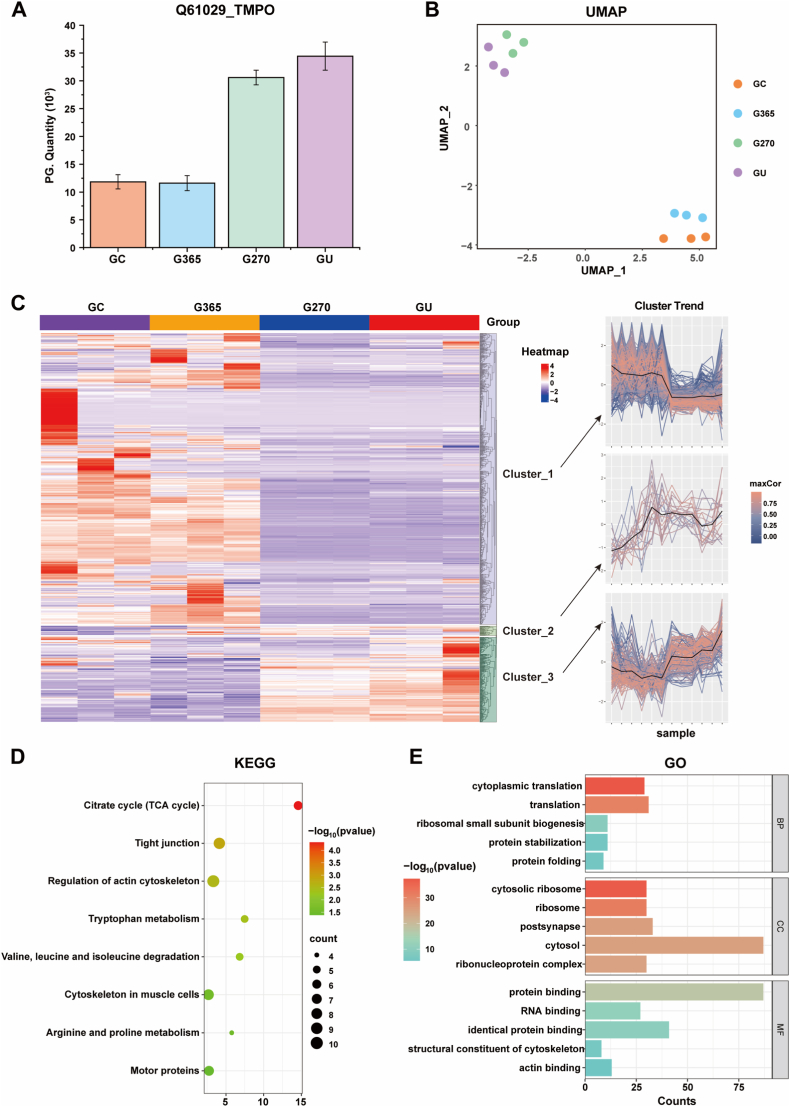
Proteomic analyses of rat BMSCs on 3D-printed implants. (A) Abundances of proteins related to nuclear protein of cell adhesion on GC, G365, G270 and GU implants. (B) UMAP analysis of the proteins on GC, G365, G270 and GU implants. (C) The heatmap of the related proteins on GC, G365, G270 and GU implants. (D) The KEGG enrichment analysis of the proteins from cluster 3 of (C). (E) The GO enrichment analysis of the proteins from cluster 3 of (C).

Proteomic analysis was performed on cells cultured under GC, G365, G270, and GU conditions to elucidate the differential protein expression patterns and their potential biological implications. The expression level of the nuclear protein TMPO gradually increased across the GC, G365, G270, and GU samples (Fig. 8A). The upregulation of TMPO may enhance cell adhesion capabilities or modulate the expression and activity of adhesion-related proteins. Additionally, it could contribute to the maintenance of cytoskeletal stability, participate in cytoskeletal assembly and reorganization, and strengthen the connection between the nucleus and the cytoskeleton. The results of Uniform Manifold Approximation and Projection (UMAP) analysis were presented to clearly delineate the four groups (GC, G365, G270, and GU) into distinct clusters, indicating good separation and discrimination among the samples (Fig. 8B). The tight clustering of data points within each group further confirmed the high consistency and reproducibility within each group. Notably, the separation between the GC/G365 group and the G270/GU group was more pronounced, with the GC and GU groups showing the most distinct separation. This suggested significant differences in the feature space between the GC and GU groups, implying that they could possess distinct biological characteristics or functions. The heatmap was depicted and revealed that the protein expression profiles of the three groups could be distinctly clustered into three clusters (Fig. 8C). Specifically, the proteins in Cluster 3 exhibited a marked increase in expression from the GC to the GU group.

**Fig. 9:**
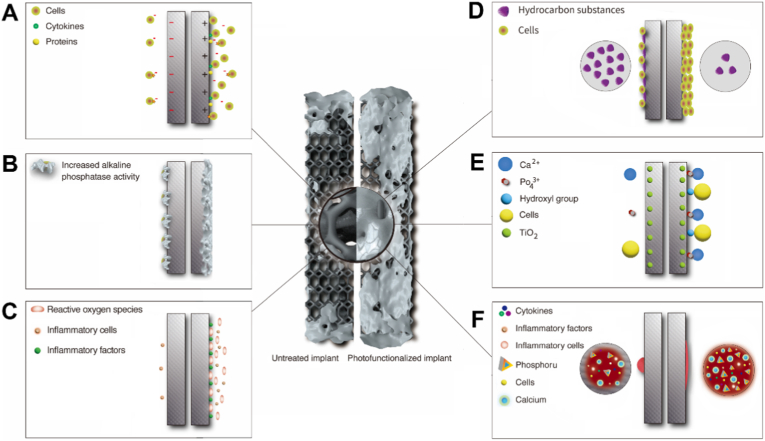
The PMO mechanism: Comparison between untreated (left schematics) and photofunctionalized (right schematics) implants. (A) Increased hydrophilicity. (B) Decreased content of heterogeneous organic matter. (C) Attraction of charges on the surface caused by (D)Functional groups such as hydroxyl and phosphates appear on the surface. (E) Biological effects: The generation of reactive oxygen species on the implant surface accelerates the process of inflammation. (F) Increased activity of surface osteoblasts.

To further explore the functional significance of these proteins, we conducted Kyoto Encyclopedia of Genes and Genomes (KEGG) and Gene Ontology (GO) functional analyses on the proteins in Cluster 3. KEGG enrichment analysis indicated that the proteins in Cluster 3 were primarily involved in several key biological processes, including the tricarboxylic acid (TCA) cycle, tight junctions, cytoskeletal components of muscle cells, regulation of the actin cytoskeleton, tryptophan metabolism, valine/leucine/isoleucine degradation, arginine and proline metabolism, and motor proteins (Fig. 8D). These functional enrichments suggested that the proteins in Cluster 3 played crucial roles in cellular energy metabolism, cytoskeletal dynamics, and cell adhesion-related pathways, highlighting the synergistic relationship between cytoskeletal and adhesion functions. In the GO analysis (Fig. 8E), the proteins in Cluster 3 were found to be involved in multiple critical biological processes, cellular components, and molecular functions. Specifically, in the biological process (BP) category, these proteins participated in essential functions such as protein stabilization and protein folding, which were vital for maintaining the normal structure and function of cellular proteins. In terms of cellular components (CC), the proteins were predominantly enriched in the cytosolic ribosome and ribosome, indicating their potential role in protein synthesis. Regarding molecular functions (MF), these proteins exhibited a variety of functions, including protein binding, RNA binding, identical protein binding, structural constituent of the cytoskeleton, and actin binding. The synergistic action of these functions likely played a key role in osseointegration on 3D-printed implants, and these implants could mediate structural maintenance, signal transduction, and metabolic regulation of rat BMSCs, which may provide important insights into the roles of these proteins in osteogenesis via adhesion-cytoskeleton-nuclear mechanotransduction (Fig. 9).

## 4 Discussion

Biomaterial-based implants are widely applied in large segmental bone defect repair for efficient bone regeneration [25]. For the UV surface treatment of titanium alloy implants, the irradiation standard hasn't been developed well in the UV surface treatment [26]. Although some studies have addressed factors such as wavelength, intensity, and power, the osteogenic effects of photofunctionalization aren't compared under different light conditions, particularly for 3D-printed porous titanium implants [27]. In the absence of comparative studies between single- and multi-wavelength UV photofunctionalization, the synergistic effect of using UV light with different wavelengths on osteogenesis remains unknown [[28], [29], [30]]. The removal of hydrocarbons from the titanium surface is the intrinsic process, which contends that the reversal of biological aging is the main cause of the osteogenic impact of photofunctionalization [31,32]. Nevertheless, the in vitro and in vivo studies show that the osteogenic impact is not exactly proportionate to hydrocarbon clearance, suggesting that a number of factors affect this process [[33], [34], [35]]. Osteogenesis is more dependent on the radiation energy (mW/cm^2^) that the implant surface receives than on the photon energy (eV) generated by the light source [[36], [37], [38]]. The energy attenuation caused by media (e.g., dust or other substances) on the light path must be considered in practical applications [39]. Moreover, the osteogenic enhancement ability of nonthermal atmospheric-pressure plasma treatment, whose hydrocarbon clearance ability is stronger than that of UV light, is nearly identical to that of UV treatment [40]. Wettability is considered a crucial factor affecting osteogenesis [[41], [42], [43]]. No difference in wettability was observed between the G270 and G365 groups in this study; however, the GU group demonstrated a significant increase in wettability and also in osteogenesis. Hydrocarbons, surface composition, and surface roughness are some of the variables that affect wettability [44]. Overall, optimizing the wettability can improve the osteogenic efficiency of 3D-printed implants [45]. We developed the 3D-printed implants which showed the hierarchical surface morphogenesis to determine their hydrophilicity, thus promoting cell adhesion and spreading behaviors (Fig. 1). 3D-printed Ti6Al4V implants were treated by UV photon functionalization to affect their carbon content, crystal structure and chemical components via both UV photon joint impact (Fig. 2).

According to our results, the GU group's BICR and ALP activity were higher than those of the G365 and G270 groups. This finding indicated that multi-wavelength UV irradiation can improve the osteogenic activity (Fig. 5). The energy of 365 nm UV light was higher than that of 270 nm UV light. The results indicated no substantial difference in BI between the GU and G270 groups, while the G365 group exhibited a significantly reduced BI (p < 0.05). This suggested that BI was influenced by the wavelength (270 nm) of UV radiation rather than its energy. As opposed to the G270 (p < 0.05) and G365 (p < 0.01) groups, the GU group's BICR was significantly greater, and the G270 group's was significantly higher than the G365 group's (p < 0.01). This suggested that UVA and UVC irradiation worked in concert to promote bone-implant binding, and that the wavelength (270 nm) was more important for BICR than UV energy. According to earlier research, UVC irradiation is crucial for the stimulation of osteogenesis on titanium surfaces, but UVA irradiation has little impact on this process. However, our study demonstrated the synergistic effect of UV-AC on osteogenesis. The underlying mechanism may be that the higher energy level of the light source in the GU group or that photons from different wavelength bands strike the titanium alloy surface [[46], [47], [48], [49]]. Furthermore, the GU group demonstrated significantly earlier bone formation than the G270 and G365 groups. This provides a physiological basis for early mechanical stability and is consistent with the biomechanical stability results [[50], [51], [52], [53], [54]]. We suggest defining the osteogenesis enhancement activity of implants by UV light as the “photomodified osteogenic (PMO) effect” (Fig. 9). BICR, BI, and mechanical stability (maximum push-out force in the experiment) are proven to evaluate the PMO effect and comprehensively assess the level of bone integration [[55], [56], [57]].

Biological mechanotransduction plays a crucial role in regulating osseointegration on 3D-printed implants [58,59]. In the force-sensing process, these biophysical stimuli are transmitted from extracellular microenvironment into cells to manage osteocyte behaviors, thus promoting bone regeneration and repair [60]. Cell adhesion proteins participate in the force-sensing mechanotransduction [[61], [62], [63], [64]]. Integrin can interact with extracellular matrices to induce the formation of focal adhesion (vinculin and talin) [[65], [66], [67]]. This adhesion behavior can transfer external force from adjacent cells or microenvironment to actin and myosin cytoskeleton to activate FAK signaling molecules, thereby inducing MAPK and YAP/TAZ signaling pathways for bone tissue repair [68,69]. The heterogeneity of focal adhesion tension can direct the phosphorylation of FAK molecules to achieve adhesion-force linear coupling for cell migration and differentiation [[70], [71], [72], [73]]. These mechanical signals will were consistent with the YAP, ERK1/2, Wnt and Notch pathways to promote osteogenic activity of BMSCs [[74], [75], [76]]. Cell nanomechanics of 3D-printed implants was induced via adhesion-cytoskeleton-nuclear coupling to stimulate integrin and vinculin expressions, which reassembled actin and myosin cytoskeletons to regulate nuclear YAP and LaminA/C force-sensing mechanotransduction for bone tissue repair (Fig. 4). Force-sensing mechanotransduction revealed the underlying mechanism of osseointegration on 3D-printed implants (Fig. 7). Further, proteomic analysis revealed protein binding, RNA binding, identical protein binding, structural constituent of the cytoskeleton, and actin binding to affect synergistic osseointegration on 3D-printed implants. Therefore, the 3D-printed implants could manage the osseointegration due to excellent biocompatibility, wettability and chemical composition through the adhesion-cytoskeleton-nuclear coupling of force-sensing mechanotransduction.

## 5 Conclusions

The 3D-printed Ti6Al4V implants were developed to highlight the combined osteogenic effects of UV light at various wavelengths and clarify the underlying mechanisms via force-sensing mechanotransduction. These implants provided the theoretical frameworks for enhancing osseointegration through synergistic surface modifications of 3D-printed titanium alloys utilizing multi-wavelength UV photofunctionalization. Mercury lamps were the most common light-emitting media for photofunctionalization; however, they emitted line spectrum and wide-spectrum UV light and had low irradiation efficiency. By contrast, the used LEDs launched a narrow band of light with high irradiation efficiency. In addition, they were a point light source, which offered more control over the spatial distribution of light compared to mercury lamps. Further research was necessary to verify the safety and effectiveness of this technique in clinical trials and to explore the effects of radiation intensity on osteogenesis. It may provide some useful information for optimal osseointegration by different combinations of UV irradiation wavelengths based on nuclear force-sensing mechanotransduction.

## CRediT authorship contribution statement

**Chuan Yin:** Writing – original draft, Methodology, Investigation, Formal analysis, Data curation, Conceptualization. **Yuan Fang:** Writing – original draft, Methodology, Investigation, Data curation. **Xiaodong Sun:** Methodology, Investigation, Formal analysis. **Zehao Jing:** Writing – review & editing. **Jingke Fu:** Methodology. **Lin Sun:** Formal analysis. **Yan Hou:** Investigation. **Eon-Bee Lee:** Writing – review & editing. **Teng Zhang:** Writing – review & editing, Supervision, Resources, Conceptualization. **Yongtao Wang:** Writing – review & editing, Writing – original draft, Supervision, Resources, Methodology, Conceptualization. **Yongqiang Hao:** Writing – review & editing, Supervision, Resources.

## Declaration of competing interest

The authors declare that they have no known competing financial interests or personal relationships that could have appeared to influence the work reported in this paper.

## Data Availability

Data will be made available on request.
