# Supplementary material for: Dual-wavelength UV photofunctionalization of 3D-printed Ti6Al4V porous bone implant enhances osseointegration via adhesion–cytoskeleton–nuclear mechanotransduction
Source: Mater Today Bio. 2025 Nov 19;35:102581. doi: 10.1016/j.mtbio.2025.102581 (PMC12681753; doi:10.1016/j.mtbio.2025.102581)
Supplement: Multimedia component 1 [file mmc1.doc]

**Dual‑wavelength UV photofunctionalization of 3D‑printed Ti6Al4V porous bone implant enhances osseointegration via adhesion–cytoskeleton–nuclear mechanotransduction**

Chuan Yin^a,b,1^,*, Yuan Fang^c, 1^, Xiaodong Sun^d,1^, Zehao Jing^e^, Jingke Fu^a,b^, Lin Sun^a,b^, Yan Hou^d^, Eon-Bee Lee^f^, Teng Zhang^g^,*, Yongtao Wang^d^,*, Yongqiang Hao^a,b^,*

^a^Shanghai Key Laboratory of Orthopaedic Implants, Department of Orthopaedic Surgery, Shanghai Ninth People’s Hospital, Shanghai Jiao Tong University School of Medicine, Shanghai 200011, China

^b^Shanghai Engineering Research Center of Innovative Orthopaedic Instruments and Personalized Medicine, Clinical and Translational Research Center for 3D Printing Technology, Shanghai 200011, China.

^c^Department of Plastic and Reconstructive Surgery, Shanghai Ninth People’s Hospital, Shanghai Jiao Tong University School of Medicine, Shanghai 200011, China

^d^School of Medicine, Shanghai University, Shanghai 200444, China

^e^Department of Orthopaedics, Peking University Third Hospital, Beijing 100191, China

^f^Department of Aquatic Life Medicine, Pukyong National University, Busan 48513, South Korea

^g^Department of Orthopedics, Qilu Hospital of Shandong University, Shandong University, Jinan 250012, China

^1^These authors contributed equally to this study.

*Corresponding authors:

Chuan Yin, E-mail: yinchuan123@pku.org.cn

Teng Zhang, E-mail: zhangtengdoc@163.com

Yongtao Wang, E-mail: yongtao_wang@shu.edu.cn

Yongqiang Hao, E-mail: sh9ychyq@163.com


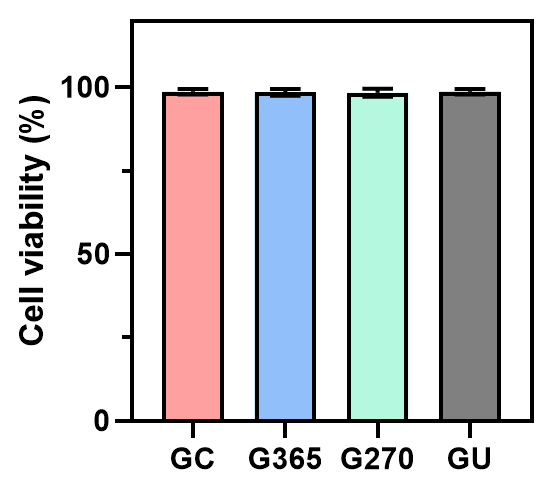


**Fig. S1.** Cell survival ability on GC, G365, G270 and GU implants.


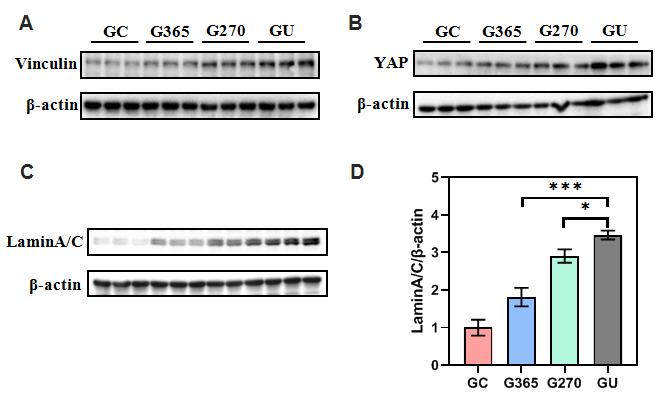


**Fig. S2.** (A) WB analysis of vinculin protein of rat BMSCs on the 3D printed implants. (B) WB analysis of YAP protein of rat BMSCs on the 3D printed implants. (C) WB analysis of LaminA/C protein of rat BMSCs on the 3D printed implants. (D) Expression level of LaminA/C protein on GC, G365, G270, and GU implants. The data present mean ± SD, n = 3, ***p* < 0.01, ****p* < 0.001.


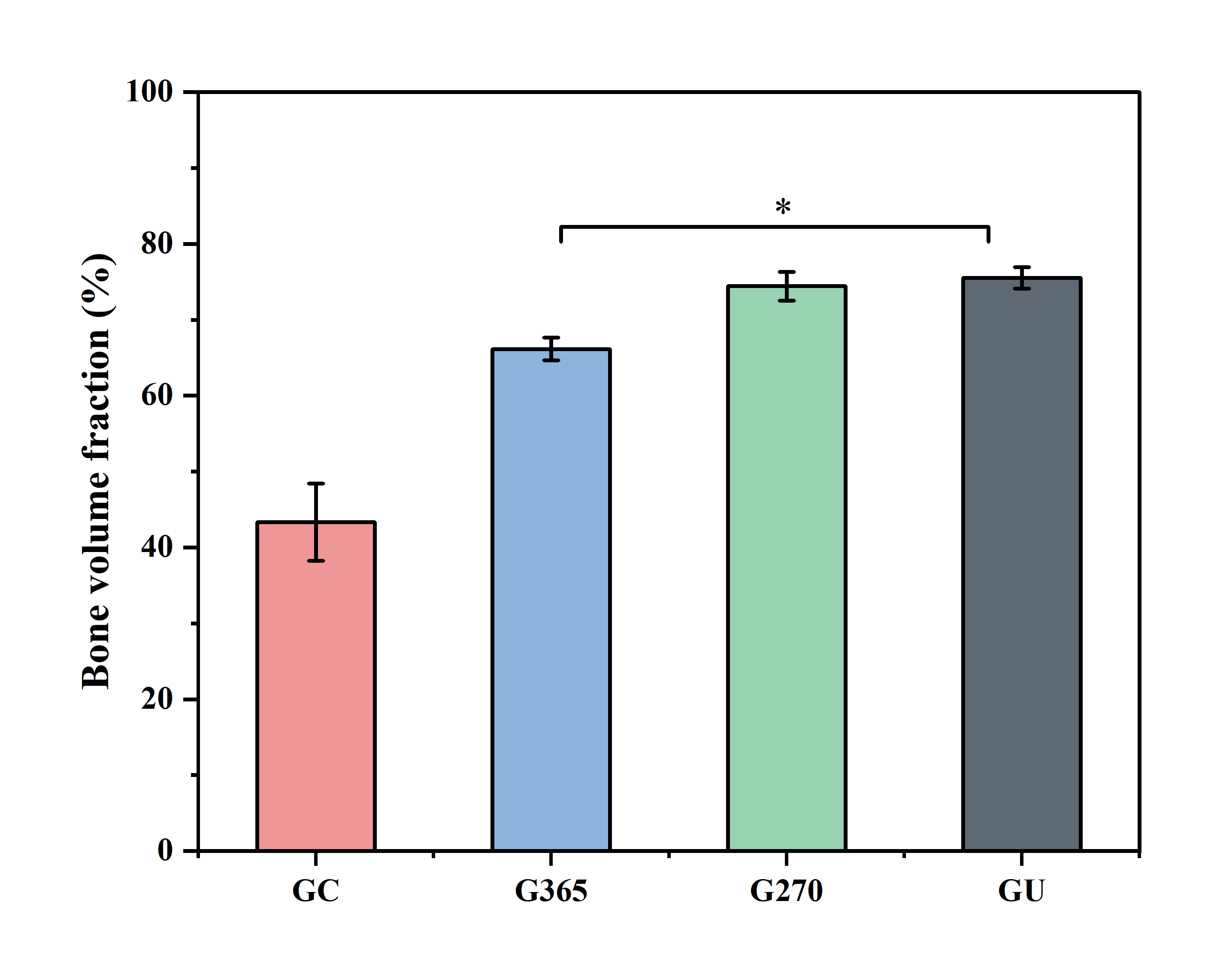


**Fig. S3.** Intraporous bone fraction of the GC, G365, G270, and GU implants. The data present mean ± SD, n = 5.


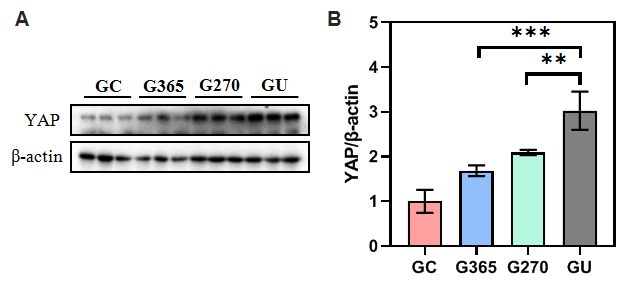


**Fig. S4.** (A) WB analysis of YAP protein of rat BMSCs on the 3D printed implants. (B) Expression level of YAP protein on GC, G365, G270, and GU implants. The data present mean ± SD, n = 3, ***p* < 0.01, ****p* < 0.001.
